# Better cardiovascular health is associated with slowed clinical progression in autosomal dominant frontotemporal lobar degeneration variant carriers

**DOI:** 10.1002/alz.14172

**Published:** 2024-09-06

**Authors:** Anna M. VandeBunte, Hyunwoo Lee, Emily W. Paolillo, Ging‐Yuek Robin Hsiung, Adam M. Staffaroni, Rowan Saloner, Carmela Tartaglia, Kristine Yaffe, David S. Knopman, Eliana Marisa Ramos, Katya Rascovsky, Andrea C. Bozoki, Bonnie Wong, Kimiko Domoto‐Reilly, Allison Snyder, Peter Pressman, Mario F. Mendez, Irene Litvan, Julie A. Fields, Douglas R. Galasko, Ryan Darby, Joseph C. Masdeu, Maria Belen Pasqual, Lawrence S. Honig, Nupur Ghoshal, Brian S. Appleby, Ian R. Mackenzie, Hilary W. Heuer, Joel H. Kramer, Adam L. Boxer, Leah K. Forsberg, Brad Boeve, Howard J. Rosen, Kaitlin B. Casaletto, the ALLFTD Consortium

**Affiliations:** ^1^ Department of Neurology University of California, San Francisco, Memory and Aging Center San Francisco California USA; ^2^ Department of Psychology Palo Alto University Palo Alto California United States; ^3^ Division of Neurology UBC Hospital University of British Columbia Vancouver British Columbia Canada; ^4^ Tanz Centre for Research in Neurodegenerative Diseases Division of Neurology Department of Medicine University of Toronto Toronto Ontario Canada; ^5^ Department of Neurology Mayo Clinic Rochester Minnesota USA; ^6^ David Geffen School of Medicine at UCLA UCLA Semel Institute for Neuroscience and Human Behavior Los Angeles California USA; ^7^ Department of Neurology University of Pennsylvania Philadelphia Pennsylvania USA; ^8^ Department of Neurology University of North Carolina Chapel Hill North Carolina USA; ^9^ Harvard Massachusetts General Hospital Frontotemporal Disorders Unit Charlestown Massachusetts USA; ^10^ Department of Neurology University of Washington Seattle Washington USA; ^11^ National Institute of Neurological Disorders and Stroke Bethesda Maryland USA; ^12^ Department of Neurology University of Colorado School of Medicine Aurora Colorado USA; ^13^ David Geffen School of Medicine at UCLA Reed Neurological Research Center Los Angeles California USA; ^14^ San Diego Department of Neurosciences University of California, San Diego La Jolla California USA; ^15^ Department of Neurology Vanderbilt University Nashville Tennessee USA; ^16^ Houston Methodist Neurological Institute Houston Texas USA; ^17^ Department of Neurology Irving Medical Center Columbia University New York New York USA; ^18^ Department of Neurology St. Louis School of Medicine Washington University St. Louis Missouri USA; ^19^ Department of Neurology Case Western Reserve University Cleveland Ohio USA; ^20^ Department of Pathology and Laboratory Medicine University of British Columbia Vancouver British Columbia Canada

**Keywords:** aging, cardiovascular health, frontotemporal dementia, genetic dementia, Life's Simple 7, lifestyle behaviors, modifiable risk, neuropsychology

## Abstract

**INTRODUCTION:**

Cardiovascular health is important for brain aging, yet its role in the clinical manifestation of autosomal dominant or atypical forms of dementia has not been fully elucidated. We examined relationships between Life's Simple 7 (LS7) and clinical trajectories in individuals with autosomal dominant frontotemporal lobar degeneration (FTLD).

**METHODS:**

Two hundred forty‐seven adults carrying FTLD pathogenic genetic variants (53% asymptomatic) and 189 non‐carrier controls completed baseline LS7, and longitudinal neuroimaging and neuropsychological testing.

**RESULTS:**

Among variant carriers, higher baseline LS7 is associated with slower accumulation of frontal white matter hyperintensities (WMHs), as well as slower memory and language declines. Higher baseline LS7 associated with larger baseline frontotemporal volume, but not frontotemporal volume trajectories.

**DISCUSSION:**

Better baseline cardiovascular health related to slower cognitive decline and accumulation of frontal WMHs in autosomal dominant FTLD. Optimizing cardiovascular health may be an important modifiable approach to bolster cognitive health and brain integrity in FTLD.

**Highlights:**

- Better cardiovascular health associates with slower cognitive decline in frontotemporal lobar degeneration (FTLD).
- Lifestyle relates to the accumulation of frontal white matter hyperintensities in FTLD.
- More optimal cardiovascular health associates with greater baseline frontotemporal lobe volume.
- Optimized cardiovascular health relates to more favorable outcomes in genetic dementia.

## 1 BACKGROUND

Of the 12 modifiable factors deemed most important for reducing dementia risk, half are directly tied to cardiovascular and cardiometabolic health.^1^ Converging epidemiologic and clinical trial data demonstrate a robust association between indicators of cardiovascular risk and late‐life all‐cause dementia and Alzheimer's disease (AD).^2^, ^3^ For instance, recent results from the SPRINT‐MIND randomized controlled trial demonstrated that intensive blood pressure lowering, compared to standard care, led to a lower incidence of mild cognitive impairment over a 4‐year period.^4^, ^5^ Even in midlife, poor cardiovascular health associates with increased microstructural brain changes and accelerated age‐related cognitive decline.^6^, ^7^ Epidemiological work has estimated that a 10% reduction in prevalence of cardiovascular risk indicators could prevent > 9 million cases of AD worldwide by 2050.^8^, ^9^ Despite strong evidence supporting the importance of cardiovascular health for bolstering cognitive reserve and reducing risk of dementia, it is still unclear whether cardiovascular health is clinically relevant in the face of underlying high genetic risk and other forms of dementia, such as frontotemporal dementia.

Frontotemporal lobar degeneration (FTLD) is a progressive neurogenerative disease that results in behavioral, cognitive, and motor dysfunction and has a strong genetic predisposition with ≈ 40% of cases estimated to be familial.^10^ Despite being among the most common causes of dementia before age 65, the role of cardiovascular health in FTLD‐related clinical progression has been understudied. One study reported a cross‐sectional association between lower body mass index, but not smoking history, and reduced risk of sporadic frontotemporal dementia.^11^ From a neuroanatomical perspective, cerebrovascular disease also demonstrates a predilection for the frontal lobes and subcortical white matter; given these areas closely overlap with FTLD‐vulnerable regions, further work is needed to evaluate associations between FTLD and cardiovascular disease.^12^, ^13^ To date, the lack of disease‐modifying treatments to target the difficult and pervasive symptoms of FTLD further underscores the importance of identifying modifiable risk factors that could mitigate the clinical expression of FTLD.

Examining cardiovascular risk in individuals with high genetic vulnerability to dementia provides a unique opportunity to assess the impact of modifiable lifestyle factors on brain health outcomes even among those with highly penetrant pathogenic variants, including autosomal dominant FTLD. One study examining adults with cerebral autosomal dominant arteriopathy with subcortical infarcts and leukoencephalopathy (CADASIL), an autosomal dominantly inherited disease defined by cerebrovascular injury, demonstrated associations between higher cardiovascular risk scores and several adverse neuroimaging markers (e.g., hippocampal atrophy, cerebral small vessel disease burden).^14^ While this finding cannot support causality, it suggests that maintenance of heart health associates with better clinical outcomes, even among individuals at the highest risk for cerebrovascular disease.

The current study examined the role of cardiovascular health on clinical outcomes in individuals with autosomal dominant FTLD. We used Life's Simple 7 (LS7), a composite metric of cardiovascular health previously associated with reduced dementia risk.^15^ We examined how baseline levels of LS7 associated with longitudinal cognitive and brain health trajectories among autosomal dominant FTLD variant carriers and non‐carrier controls from an international, multi‐site study. To determine the characteristics of this relationship, we probed the impact of FTLD variant carrier status (yes/no), genotype, and the individual LS7 cardiovascular health indicators most strongly predictive of clinical trajectories. Last, we examined the same associations in only asymptomatic FTLD variant carriers to examine the association between optimal cardiovascular health with preclinical cognitive and brain health trajectories without the potential confound of FTLD behavioral symptoms.

**RESEARCH IN CONTEXT**

1. **Systematic review**: The existent literature on the role of cardiovascular health in frontotemporal lobar degeneration (FTLD) clinical progression is understudied. While there are data suggesting a link between cardiovascular‐related health factors and FTLD, these publications have been limited by (1) only examining indirect indicators of cardiovascular health and (2) cross‐sectional designs that cannot rule out that associations were confounded by prodromal FTLD symptoms. These relevant citations are appropriately cited.
2. **Interpretation**: Our findings suggest that examining modifiable behaviors and cardiovascular health states is an important approach that may support cognitive health and brain integrity, even in genetic forms of dementia.
3. **Future directions**: This article is among the first to comprehensively examine several facets of cardiovascular health (e.g., lifestyle) in individuals with FTLD. Future analyses integrating objective monitoring of cardiovascular health states (e.g., wearables) would help to more precisely understand optimal levels of cardiovascular health necessary for brain health trajectories in FTLD.

## 2 METHODS

### 2.1 Participants

Participants included family members affected by the genetic forms of FTLD and enrolled in the Advancing Research and Treatment in Frontotemporal Lobar Degeneration (ARTFL) and Longitudinal Evaluation of Familial Frontotemporal Dementia (LEFFTDS) and Longitudinal Frontotemporal Degeneration (ALLFTD) study based in the United States and Canada. Participants were included based on completion of neurobehavioral outcomes and at least five of the seven cardiovascular health indicators included in the LS7 score. Using these criteria, we included 247 individuals carrying a pathogenic variant of *MAPT, GRN*, or *C9orf72* (128 asymptomatic, Global Clinical Dementia Rating + National Alzheimer's Coordinating Center Behavior and Language Domains [CDR+NACC FTLD] = 0) and 189 non‐carrier controls (Global CDR+NACC FTLD = 0; Table 1). Of the symptomatic FTLD pathogenic carriers (47% of the cohort), a variety of clinical syndromes were present (Table S1 in supporting information). All genetic testing was completed in the same laboratory at the University of California, Los Angeles using standardized methods, as previously described.^16^ ALLFTD is an ongoing longitudinal study with approximately annual visits.

**TABLE 1:** Clinical and demographic characteristics of baseline sample.

				Independent samples *t* test	
	Whole sample (*N* = 436)	FTLD variant carrier (*n* = 247)	Non‐carrier (*n* = 189)	*t*(df)	*p* value
Years in study (%, *n*)				–	–
1	100% (436)	100% (247)	100% (189)		
2	67.4% (294)	100% (134)	84.6% (160)		
3	42.2% (184)	34.8% (86)	51.8% (98)		
4	19.0% (83)	14.6 (36)	24.9% (47)		
5	8.7% (38)	5.7% (14)	12.7% (24)		
6	3.4% (15)	2.0% (5)	5.3% (10)		
7	0.7% (3)	0.4% (1)	1.1% (2)		
Age	48.5 (14.4)	49.8 (14.9)	46.7 (13.3)	2.26 (424.5)	0.02^a^
Sex, % female (*n*)	57.6% (251)	51.8% (128)	65.1% (123)	–	–
Education	15.5 (2.4)	15.3 (2.5)	15.8 (2.4)	–1.84 (412.3)	0.03^a^
Genotype^b^ (%, *n*)					
*C9orf72*	–	57.5% (142)	–	–	–
*GRN*		19.0% (47)			
*MAPT*		22.3% (55)			
CDR+NACC FTLD‐SB	1.89 (4.09)	3.34 (4.98)	0.00 (0.00)	10.55 (246.0)	0.00^a^
CDR+NACC FTLD, Global (%, *n*)					
= 0		53.0% (131)^b^	100% (189)	–	–
= 0.5		14.2% (35)			
≥ 1		32.8% (81)			
Life's Simple 7 (sum 0–14)	8.58 (2.18)	8.45 (2.27)	8.75 (2.14)	−1.42 (415.4)	0.16
Memory (*z* score)^c^	−0.46 (1.14)	−0.82 (1.29)	0.00 (0.69)	−8.49 (383.1)	0.00^a^
Executive functioning (*z* score)^c^	−0.79 (1.47)	−1.25 (1.73)	−0.18 (0.67)	−8.84 (329.7)	0.00^a^
Language (*z* score)^c^	−0.73 (1.56)	−1.22 (1.85)	−0.10 (0.68)	−8.69 (321.3)	0.00^a^
Frontotemporal lobe gray matter volume (voxels, 1 cm^3^)	155,812.7 (21,593.9)	151,762.4 (24,340.8)	160,350.15 (17,002.8)	−3.46 (265.2)	0.00^a^
Frontal white matter hyperintensity volume (mm^3^)	564.58 (1314.52)	667.59 (1309.33)	458.36 (1316.51)	1.28 (257.7)	0.20

*Note*: Mean (SD) or % (*n*) reported.

Abbreviations: CDR plus NACC FTLD‐SB, Clinical Dementia Rating Dementia Staging Instrument PLUS National Alzheimer's Coordinating Center (NACC) Behavior and Language Domain, Sum of Boxes; FTLD, frontotemporal lobar degeneration; SD, standard deviation.

^a^ Statistically significant at 0.05.

^b^ Three participants with both *C9orf72* and *GRN* variants were excluded for the subgroup analyses and in analyses with asymptomatic carriers only.

^c^ *z* scores on these tests represent performances compared to healthy non‐carrier controls.

The study was approved by the appropriate institutional review boards and is conducted in accordance with the latest Declaration of Helsinki, including written informed consent from all participants.

### 2.2 Baseline cardiovascular health

Participants were screened for LS7 cardiovascular health indicators during a comprehensive neurobehavioral and clinical interview. LS7 components included self‐reported physical activity (Physical Activity Scale for the Elderly) and diet, body mass index (calculated from baseline height and weight), and prior or current smoking, hypertension, diabetes, and hypercholesterolemia statuses (Table 2). Each LS7 component was assigned poor, intermediate, or ideal scores following previously established procedures,^17^ and a total (14 point) score was generated based on methods adapted from the REGARDS study.^18^ Higher total LS7 scores indicate more optimal cardiovascular health.

**TABLE 2:** Individual Life's Simple 7 factors in baseline sample.

	Whole sample (*n* = 436)	FTLD variant carrier (*n* = 247)	Non‐carrier (*n* = 189)
Life's Simple 7 (sum 0–14)	8.58 (2.18)	8.45 (2.27)	8.75 (2.14)
Body mass index	*n* = 386 27.47 (6.39)	*n* = 228 26.59 (6.40)	*n* = 158 28.76 (7.23)
Hypertension history (%, *n*)	*n* = 436	*n* = 247	*n* = 189
Recent/active	14.4% (63)	17.8% (44)	10.0% (19)
Remote/inactive	<1% (4)	1.2% (3)	<1% (1)
Absent	84.6% (369)	81.0% (200)	89.4% (169)
Hypercholesteremia history (%, *n*)	*n* = 434	*n* = 246	*n* = 188
Recent/active	19.1% (83)	22.0% (54)	15.4% (29)
Remote/inactive	1.8% (8)	1.6% (4)	2.1% (4)
Absent	79.1% (343)	76.4% (188)	82.4% (155)
Diabetes history (%, *n*)	*n* = 436	*n* = 247	*n* = 189
Recent/active	11.0% (48)	4.5% (14)	4.8% (9)
Remote/inactive	<1% (1)	<1% (1)	0.0% (0)
Absent	94.5% (412)	93.9% (232)	95.2% (180)
Smoking history (%, *n*)	*n* = 429	*n* = 242	*n* = 187
Last 30 days	11.2% (48)	9.9% (24)	12.8% (24)
More than 100 in lifetime	20.7% (89)	20.2% (49)	21.4% (40)
Never smoked/less than 100 in lifetime	68.1% (292)	69.8% (169)	65.8% (123)
Diet^a^ (%, *n*)	*n* = 194	*n* = 97	*n* = 97
Poor	47.4% (92)	51.5% (50)	43.3% (42)
Intermediate	47.4% (92)	41.2% (40)	53.6% (52)
Ideal	5.1% (10)	7.2% (7)	3.1% (3)
PASE total score median (IQR)	*n* = 210 122.88 (80.9, 156.7)	*n* = 108 109.54 (62.4, 153.9)	*n* = 102 129.50 (98.9, 164.9)

*Note*: Mean (SD) or % (*n*) reported.

Abbreviations: FTLD, frontotemporal lobar degeneration; IQR, interquartile range; PASE, Physical Activity Scale for the Elderly; SD, standard deviation.

^a^ Diet score was calculated based on previously established methods.^18^

### 2.3 Brain structural outcomes

Study participants were scanned on 3T magnetic resonance imaging (MRI) scanners from GE, Siemens, or Philips, according to the standardized Alzheimer's Disease Neuroimaging Initiative (ADNI)‐3 Protocol.^19^ Images were managed and reviewed for quality by a core MRI group at the Mayo Clinic, Rochester, Minnesota. All participants except three were scanned on the same scanner at all visits (for two participants, the scanner was upgraded; the third changed sites). Details of image acquisition, processing, and harmonization have been previously described.^20^

#### 2.3.1 Gray matter volume

T1‐weighted images were processed using SPM12 software, which provided segmentations of the gray matter, white matter, and cerebrospinal fluid (CSF). For this study, frontotemporal lobe volume was estimated by summing bilateral temporal lobe and frontal lobe regions. We elected to examine frontotemporal lobar measures as our primary brain MRI outcome, as FTLD has previously been associated with changes in this region.^21^ To obtain the frontotemporal gray matter labels, the gray matter segmentations and the Desikan–Killiany standard atlas labels^22^ were linearly and then non‐linearly registered into the International Consortium for Brain Mapping space. The individual atlas labels corresponding to the frontotemporal gray matter were summed and then applied to the modulated gray matter segmentations to obtain the frontotemporal gray matter volume for each subject. Total intracranial volume (TIV) was calculated for each subject as the sum of the gray matter, white matter, and CSF segmentations.

#### 2.3.2 Frontal white matter hyperintensities

T1‐weighted images were processed using FreeSurfer software, which provided cortical reconstruction and gray matter/white matter volumetric segmentations.^23^ All FreeSurfer outputs were manually corrected for possible errors. Fluid‐attenuated inversion recovery images were processed using a Bayesian 3D convolutional neural network‐based pipeline,^24^ which provided white mattery hyperintensity (WMH) segmentations. WMH segmentations were linearly registered to the T1‐weighted space, and the above‐mentioned frontal lobar white matter labels were applied to obtain the frontal lobar WMH volumes. We elected to examine frontal lobar WMHs given existing evidence of frontal white matter degradation preferentially relating to cardiovascular disease.^12^, ^13^, ^25^ For each subject, TIV was estimated using a multi‐atlas label fusion technique, described in detail elsewhere.^26^

### 2.4 Cognitive outcomes

Participants completed cognitive measures from the third version of the National Institutes of Health NACC Uniform Data Set neuropsychological battery^27^ that includes an additional module for assessment of FTLD, and the California Verbal Learning Test, short form (CVLT‐II, SF^28^). Sample‐based *z* scores were computed using healthy non‐carrier controls to create composite scores for each cognitive domain. Regarding missing cognitive data, participants were only included in each cognitive composite score if they completed the majority of subtests. For the memory composite, participants were included if they had at least two of three subtests; for executive functioning at least three out of five; and similarly for language, they were included if they completed two of three subtests.

The executive functioning composite included the longest number of digits recalled in backward order, total completion time (in seconds) for Trail Making Test Parts A and B, and two phonemic fluency trials (generation of words beginning with the letters “F” and “L,” each in 1 minute). Measures of visual episodic memory (10‐minute delayed recall of the Benson Complex Figure Test), verbal episodic memory (Craft Story 21 Recall [Delayed] task), and verbal list recall (CVLT‐II, SF, 10‐minute delayed recall) were included in the memory composite. Finally, the language composite consisted of two category fluency trials (generation of animal and vegetable names, each in 1 minute) and an object naming test (Multilingual Naming Test).

### 2.5 CDR+NACC FTLD

The CDR+NACC FTLD^29^, ^30^ was used as a marker of functional severity. The CDR+NACC FTLD includes ratings across six functional domains captured in the traditional CDR, in addition to two new domains specific to the core clinical features of FTLD: language and behavior. Following a standardized algorithm,^29^, ^30^ the eight domain scores were summed to create a global score (0–8), while each domain was scored on a scale from 0 to 3 and summed to create a more continuous measure of symptom severity (0–24) referred to as the Sum of Boxes (CDR+NACC FTLD‐SB).

### 2.6 Statistical analyses

We first examined whether there were differences in baseline LS7 scores between FTLD variant carriers and non‐carrier controls or among genotypes (*C9orf72*, *GRN*, *MAPT*) via an independent samples *t* test or analysis of variance with Tukey honestly significant difference (HSD), respectively.

#### 2.6.1 Cross‐sectional models

In FTLD variant carriers only, we examined the baseline relationships between LS7 scores and cognitive (e.g., memory, language, and executive functioning composite scores) and brain structural (e.g., frontotemporal lobe gray matter volume, frontal WMH volume) outcomes using multivariate linear regression models, covarying for age, sex, education, and CDR+NACC FTLD‐SB. Additionally, all neuroimaging models were adjusted for TIV. Frontal WMH volume was log‐transformed due to a significant positive skew. Interaction terms (LS7 x carrier status) tested the differential associations of variant carrier status on relationships between LS7 and cognitive and brain structural outcomes.

#### 2.6.2 Longitudinal models

To most optimally fit longitudinal models, only participants with at least two time points were included (*n* = 294). In FTLD variant carriers only, we examined relationships between baseline LS7 and longitudinal cognitive and brain structural trajectories by entering the interaction between baseline LS7 and time in study (years since baseline), adjusting for baseline age, sex, education, and baseline CDR+NACC FTLD‐SB. Neuroimaging models were also adjusted for baseline TIV. All models estimated subject‐specific random intercepts and a random effect of time. We next tested the moderating effect of variant carrier status (carriers/non‐carriers) by entering a three‐way interaction term (baseline LS7 x time x carrier status). Sensitivity analyses including only the FTLD variant carriers who remained asymptomatic (Global CDR+NACC FTLD = 0, *n* = 102) across the study period were conducted to inform primary prevention approaches.

Given that some of the participants included in the study did not have all the LS7 factors, we also conducted sensitivity analyses using a mean imputation approach to evaluate the robustness of significant models. To do so, we calculated the group average for each individual LS7 factor and then assigned the group average value to participants with a missing LS7 factor prior to summing a total LS7 score.

For our primary longitudinal models, we conducted follow‐up sensitivity analyses that included additional interaction terms between each demographic covariate and time to examine whether the pattern of results held.

#### 2.6.3 Post hoc models

Last, we tested post hoc models to explore the characteristics (impact of genotype or individual LS7 factors) of the significant relationships between baseline LS7 and clinical outcomes in FTLD variant carriers. First, to understand how each cardiovascular disease (CVD) indicator may be driving overall findings, individual LS7 factors were entered separately into linear mixed effects (LME) models predicting cognitive and brain structural trajectories, as above. Additionally, to evaluate the influence of genotype, we conducted cross‐sectional (multivariate regression models, baseline LS7 x genotype) and longitudinal (LME model, baseline LS7 x time x genotype) analyses that evaluated interactions between baseline LS7 and genotype (*C9orf72, GRN, MAPT*, non‐carriers) on cognitive and brain structural outcomes. To further probe these results, we also conducted the same models stratified by genotype. All analyses were adjusted for demographics and baseline CDR+NACC FTLD‐SB.

Across all models, effect sizes are reported as standardized betas and 95% confidence interval or standard error.

## 3 RESULTS

FTLD variant carriers and non‐carrier controls did not significantly differ on baseline LS7 (Table 1). When accounting for age, sex, and education level, baseline LS7 scores significantly differed by genotype (*F*_2,238 _= 3.31, *p *= 0.04). Post hoc Tukey HSD analysis revealed that omnibus group differences were driven by *C9orf72* variant carriers who had higher (better) baseline LS7 scores than *MAPT* carriers (mean difference = −0.77, *p *= 0.05). There were no other differences in baseline LS7 scores among genotypes (*P*s > 0.28).

### 3.1 Cross‐sectional

#### 3.1.1 FTLD variant carriers

**FIGURE 1:**
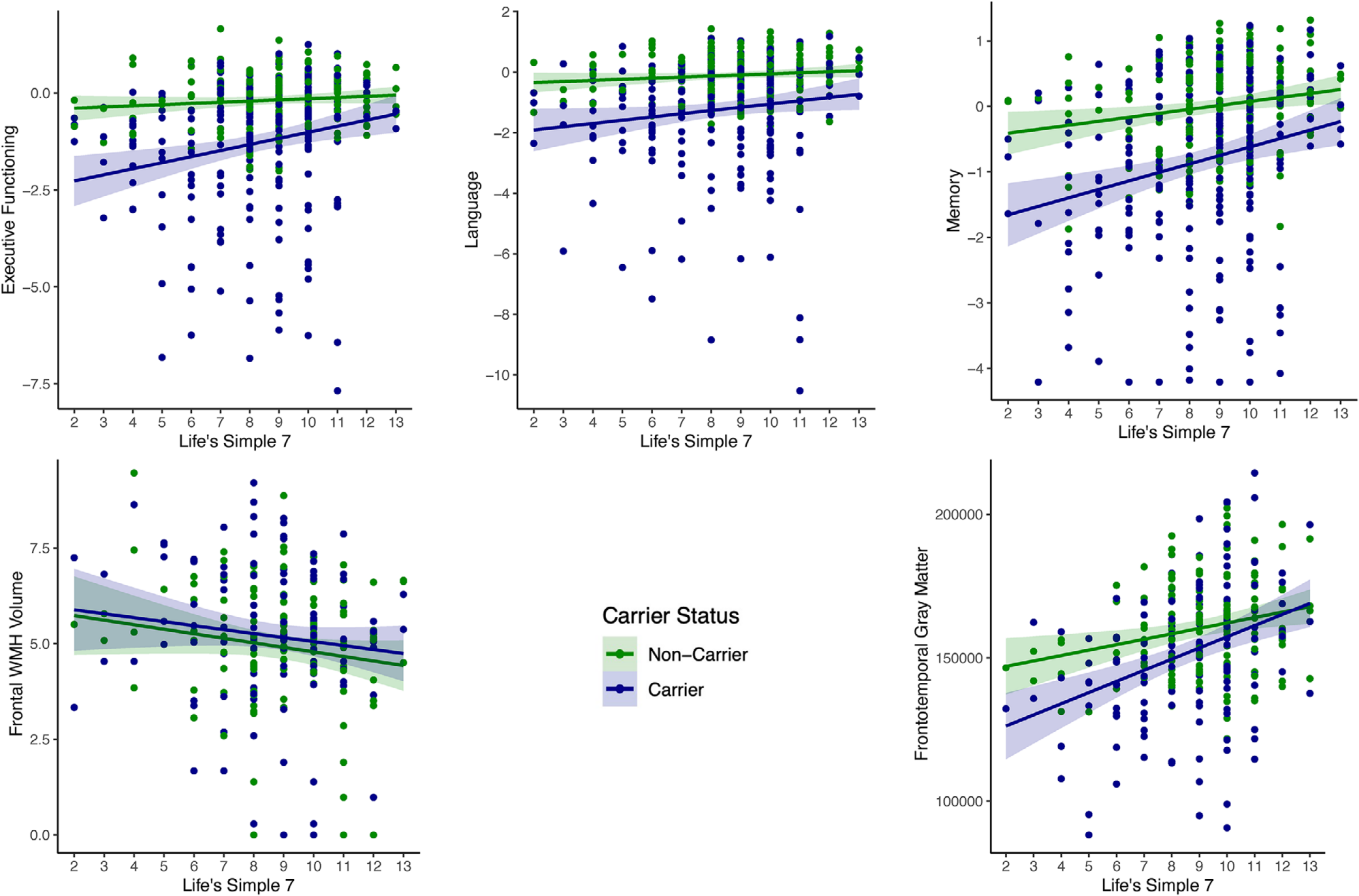
Multivariate regression interaction models (baseline LS7 x carrier status) examining associations between baseline LS7 and cognitive and brain health outcomes in FTLD variant carriers compared to healthy non‐carrier controls. FTLD, frontotemporal lobar degeneration; LS7, Life's Simple 7; WMHs, white matter hyperintensities.

Higher LS7 associated with larger gray matter volume in the frontotemporal lobe at baseline in FTLD variant carriers only (*β* = 0.14, *p *< 0.001), accounting for age, education, sex, TIV, and CDR+NACC FTLD‐SB. Baseline associations between LS7 and additional outcomes of interest (frontal WMHs, language, memory, executive functioning) did not reach statistical significance in FTLD variant carriers (Table 3; Figure 1).

**TABLE 3:** Linear regression models examining the relationship between baseline LS7 and cognitive and brain structural outcomes in FTLD variant carriers.

	Memory		Language		Executive functioning		Frontotemporal GMV		Frontal WMHs	
	*β* (95% CI)	*p* value	*β* (95% CI)	*p* value	*β* (95% CI)	*p* value	*β* (95% CI)	*p* value	*β* (95% CI)	*p* value
Age	−0.21 (−0.32, −0.11)	<0.001	0.05 (−0.07, 0.16)	0.42	−0.05 (−0.16, 0.06)	0.38	−0.43 (−0.52, −0.35)	<0.001	0.36 (0.18, 0.54)	<0.001
Education	0.15 (0.06, 0.24)	<0.001	−0.09 (−0.18, 0.01)	0.08	0.03 (−0.06, 0.12)	0.52	−0.03 (−0.10, 0.04)	0.41	−0.08 (−0.24, 0.08)	0.34
Sex	0.03 (−0.15, 0.20)	0.77	0.12 (−0.06, 0.31)	0.20	0.07 (−0.11, 0.25)	0.46	0.12 (−0.06, 0.30)	0.19	0.21 (−0.16, 0.58)	0.26
Total intracranial volume	–	–	–	–	–	–	0.53 (0.45, 0.62)	<0.001	0.09 (−0.10, 0.27)	0.36
CDR+NACC FTLD‐SB	−0.58 (−0.68, −0.48)	<0.001	−0.72 (−0.83, −0.61)	<0.001	−0.67 (−0.78, −0.57)	<0.001	−0.39 (−0.47, −0.32)	<0.001	0.31 (0.14, 0.48)	<0.001
LS7 (0–14)	−0.00 (−0.10, 0.09)	0.99	0.01 (−0.09, 0.12)	0.78	0.04 (−0.06, 0.14)	0.38	0.14 (0.06, 0.21)	<0.001	0.03 (−0.13, 0.20)	0.68

*Note*: *β* standardized beta values. For LS7, higher scores represent more optimal cardiovascular health.

Abbreviations: CDR plus NACC FTLD‐SB, Clinical Dementia Rating Dementia Staging Instrument PLUS National Alzheimer's Coordinating Center (NACC) Behavior and Language Domain, Sum of Boxes; CI, confidence interval; FTLD, frontotemporal lobar degeneration; LS7, Life's Simple 7.

#### 3.1.2 FTLD variant carriers and non‐carrier controls

To examine whether significant associations differed between FTLD variant carriers and non‐carrier controls, we tested the same models including a two‐way interaction term (baseline LS7 x carrier status). Indeed, there was a significant interaction between LS7 and carrier status on frontotemporal volume (Figure 1, Table S2 in supporting information), such that the positive association between LS7 and frontotemporal volume was stronger among FTLD variant carriers versus non‐carriers.

### 3.2 Longitudinal models

#### 3.2.1 FTLD variant carriers

We next evaluated relationships between baseline LS7 and longitudinal cognitive and brain structural trajectories in FTLD variant carriers only. Higher baseline LS7 scores are associated with slower frontal WMH accumulation and slower decline in memory and language functioning over time (Table 4). Baseline LS7 was not associated with longitudinal frontotemporal volume or executive functioning trajectories.

**TABLE 4:** Mixed effects models examining the relationship between baseline LS7 and cognitive and brain structural trajectories in FTLD variant carriers.

	Memory		Language		Executive Functioning		Frontotemporal GMV		Frontal WMHs	
	*β* (95% CI)	*p* value	β (95% CI)	*p* value	*β* (95% CI)	*p* value	*β* (95% CI)	*p* value	*β* (95% CI)	*p* value
Baseline age	−0.24 (−0.36, −0.12)	<0.001	−0.02 (−0.15, 0.12)	0.82	0.03 (−0.09, 0.15)	0.67	−0.51 (−0.61, −0.40)	<0.001	0.47 (0.26, 0.68)	<0.001
Education	0.11 (0.00, 0.21)	0.05	−0.09 (−0.21, 0.03)	0.13	0.04 (−0.07, 0.14)	0.46	−0.05 (−0.14, 0.05)	0.35	−0.17 (−0.37, 0.03)	0.11
Sex	−0.05 (−0.26, 0.15)	0.60	0.11 (−0.12, 0.35)	0.34	−0.08 (−0.28, 0.13)	0.46	0.10 (−0.17, 0.36)	0.47	−0.03 (−0.52, 0.46)	0.92
Baseline CDR+NACC FTLD‐SB	−0.50 (−0.60, −0.40)	<0.001	−0.71 (−0.82, −0.59)	<0.001	−0.63 (−0.73, −0.53)	<0.001	−0.29 (−0.37, −0.21)	<0.001	0.23 (0.05, 0.42)	0.02
Baseline total intracranial volume	–	–	–	–	–	–	0.50 (0.38, 0.63)	<0.001	−0.04 (−0.28, 0.20)	0.77
Baseline LS7 (0–14)	0.05 (−0.06, 0.17)	0.38	0.02 (−0.11, 0.14)	0.78	0.06 (−0.07, 0.19)	0.38	0.12 (0.02, 0.23)	0.02	−0.01 (−0.22, 0.20)	0.90
Time in study	0.03 (−0.03, 0.08)	0.39	−0.04 (−0.09, 0.01)	0.10	−0.04 (−0.11, 0.03)	0.24	−0.13 (−0.17, −0.10)	<0.001	0.07 (−0.02, 0.16)	0.13
Baseline LS7 x time	0.09 (0.03, 0.15)	0.01	0.07 (0.02, 0.12)	<0.001	0.04 (−0.03, 0.10)	0.30	−0.02 (−0.06, 0.01)	0.22	−0.10 (−0.19, 0.00)	0.04

*Notes*: *β* standardized beta values. For LS7, higher scores represent more optimal cardiovascular health.

Abbreviations: CDR plus NACC FTLD‐SB, Clinical Dementia Rating Dementia Staging Instrument PLUS National Alzheimer's Coordinating Center (NACC) Behavior and Language Domain, Sum of Boxes; CI, confidence interval; FTLD, frontotemporal lobar degeneration; GMV, gray matter volume; LS7, Life's Simple 7; WMHs, white matter hyperintensities.

When conducting additional sensitivity models that evaluated relationships between baseline LS7 and longitudinal cognitive (memory and language) and frontal WMH trajectories, the pattern of results remained similar (Table S3 in supporting information).

To inform primary prevention approaches and examine relationships between cardiovascular risk and clinical outcomes in FTLD variant carriers without potentially confounding FTLD symptoms, we conducted the same analyses but excluded symptomatic FTLD variant carriers. In asymptomatic FTLD variant carriers only (*n* = 102), LS7 associations showed similar effect sizes for memory (*β* = 0.11, *p *= 0.029), language (*β* = 0.10, *p *= 0.046), and frontal WMHs (*β* = −0.08, *p *= 0.15) trajectories, though the latter did not reach statistical significance.

#### 3.2.2 FTLD variant carriers and non‐carrier controls

**FIGURE 2:**
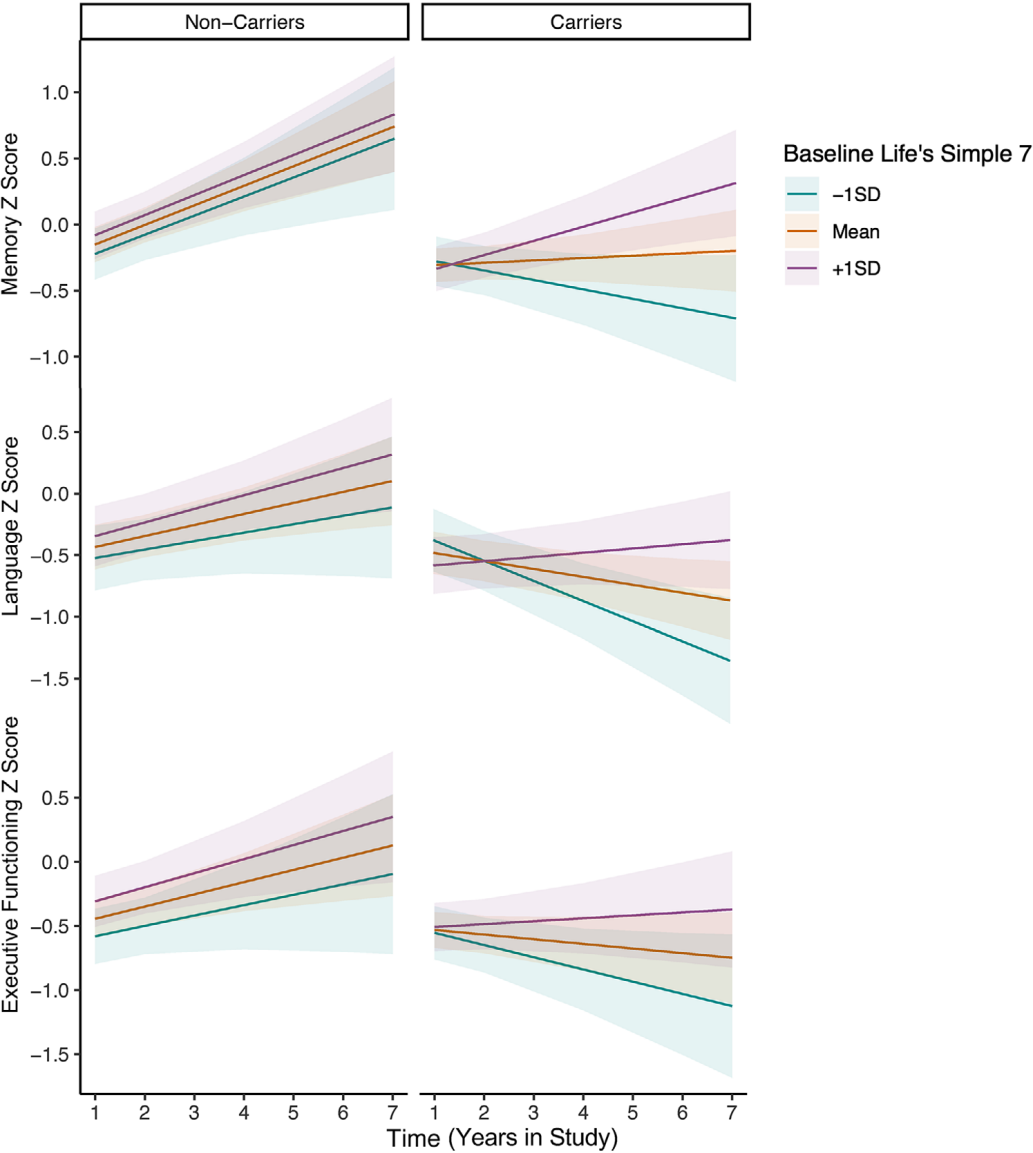
Linear mixed effects interaction models (baseline LS7 x time x carrier status) examining associations between baseline LS7 and cognitive trajectories in FTLD variant carriers compared to healthy non‐carrier controls. FTLD, frontotemporal lobar degeneration; LS7, Life's Simple 7; SD, standard deviation.

**FIGURE 3:**
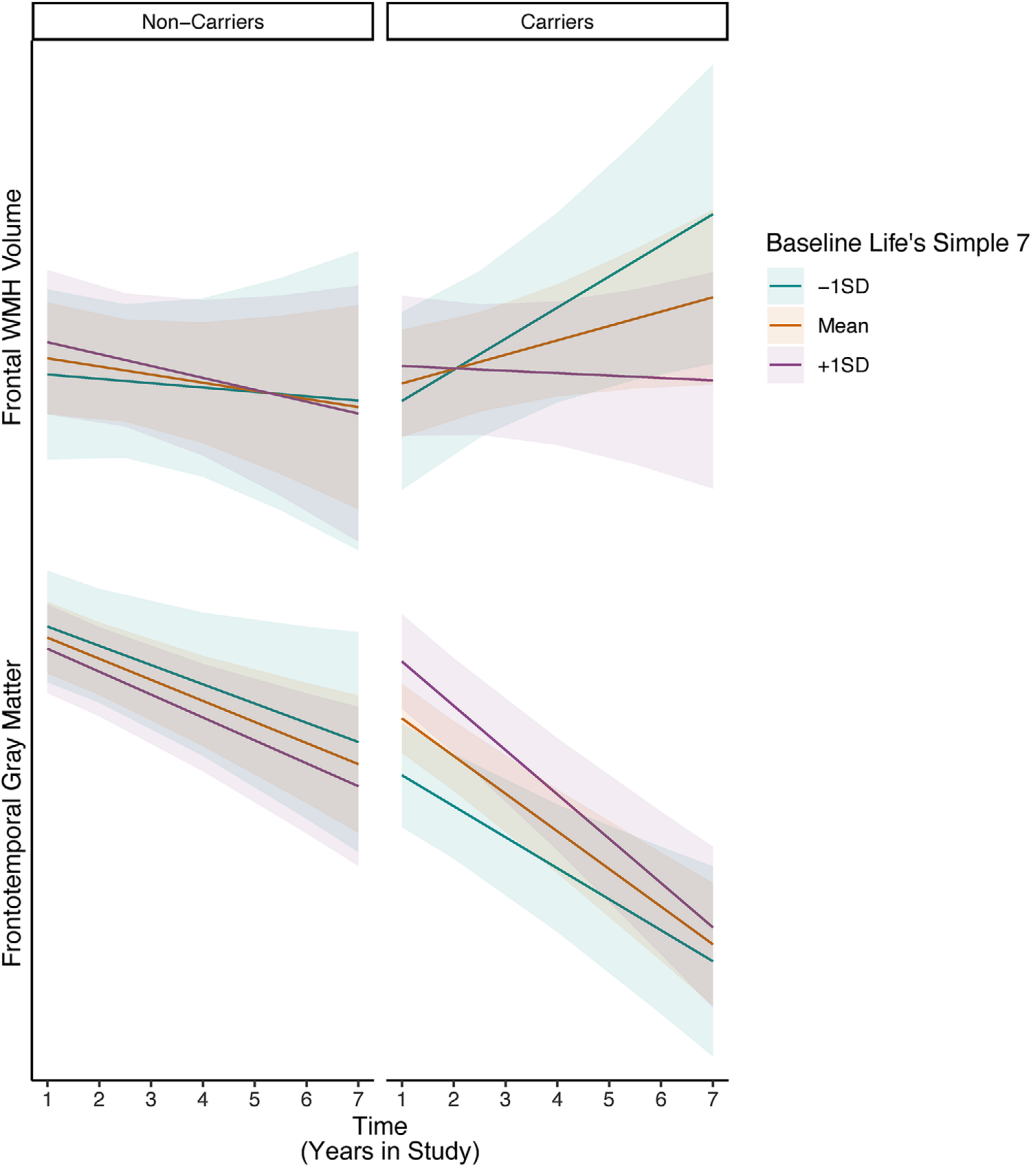
Linear mixed effects interaction models (baseline LS7 x time x carrier status) examining associations between baseline LS7 and neuroimaging trajectories in FTLD variant carriers compared to healthy non‐carrier controls. FTLD, frontotemporal lobar degeneration; LS7, Life's Simple 7; SD, standard deviation; WMH, white matter hyperintensity.

Next, we tested whether associations between baseline LS7 and clinical trajectories differed in FTLD variant carriers versus non‐carriers in the entire sample (baseline LS7 x carrier status x time). There was a statistically significant interaction between carrier status and LS7 on memory performances over time, such that the association between better baseline LS7 and slower memory decline was stronger among FTLD variant carriers versus non‐carriers (Figure 2), even when excluding symptomatic carriers (*β* = 0.13, *p *= 0.047). A sensitivity model that included additional interaction terms between each demographic covariate and time revealed the same statistically significant interaction between carrier status and LS7 on memory trajectories (Table S4 in supporting information). The interaction with carrier status did not reach statistical significance for language, executive functioning, frontotemporal volumes, or frontal WMH trajectories (Figures 2 and 3, Table S5 in supporting information).

In sensitivity models using a group average imputation for those with missing LS7 items, the overall pattern of cross‐sectional and longitudinal results remained the same.

### 3.3 Post hoc models examining individual LS7 factors and genotype

#### 3.3.1 Individual LS7 factors

To determine whether specific cardiovascular health indicators drove associations between overall LS7 scores and memory, language, and frontal WMH trajectories in FTLD variant carriers, we extracted each individual LS7 factor from the overall score. Better blood pressure, cholesterol, and body mass indices were most strongly associated with memory trajectories in FTLD variant carriers (Figure S1 in supporting information). Similarly, out of the seven LS7 indicators, more optimal blood pressure and cholesterol were also most strongly associated with language trajectories (Figure S2 in supporting information). None of the individual LS7 factors demonstrated distinct, significant associations with frontal WMH accumulation over time (Figure S3 in supporting information).

#### 3.3.2 Genotype

Cross‐sectionally, there was a significant interaction between baseline LS7 and genotype (non‐carriers set as reference group) on baseline frontotemporal gray matter volumes, such that *GRN* (*β* = 0.24, *p *= 0.005) and *MAPT* (*β* = 0.36, *p *< 0.001) carriers evidenced significantly stronger associations between better cardiovascular health and larger frontotemporal volumes at baseline compared to non‐carriers. Similarly, stratified models revealed significant relationships between LS7 and baseline frontotemporal volume only in *MAPT* (*β* = 0.25, *p *= 0.001) and *GRN* (*β* = 0.23, *p *= 0.010) variant carriers, and not in *C9orf72* (*β* = 0.06, *p *= 0.264).

Similarly, there was an interaction between baseline LS7 and genotype (non‐carriers set as reference group) on memory decline over time, such that the protective relationship was strongest in the *MAPT* carriers compared to non‐carriers (*β* = 0.16, *p *= 0.028), but did not differ for *C9orf72* (*β* = 0.09, *p *= 0.09) or *GRN* (*β* = 0.09, *p *= 0.33) variant carriers. The interaction between baseline LS7 and genotype on language and frontal WMH trajectories did not reach statistical significance (*β* range = −0.11–0.11, *p *> 0.05). However, stratified models revealed significant relationships between LS7 and memory (*β* = 0.11, *p *= 0.028) and language (*β* = 0.08, *p *= 0.022) trajectories in *MAPT* variant carriers, but not in *C9orf72* or *GRN* (*β* range = −0.07–0.07, *p *> 0.05). It is notable that *GRN* variant carriers represented the smallest sample size (*n* = 47) and evidenced the largest confidence intervals suggesting additional work is needed for more precise estimation.

## 4 DISCUSSION

We demonstrate that better cardiovascular health is associated with several cognitive and brain health outcomes in individuals with high genetic risk for FTLD, including slower declines in memory and language functioning and accumulation of frontal WMH burden. Among indicators of cardiovascular health, blood pressure and cholesterol showed the largest individual associations with cognitive trajectories. Within genotypes, the positive association of cardiovascular health on clinical trajectories was strongest in *MAPT* and *C9orf72* carriers. These are among the first data comprehensively evaluating the role of cardiovascular risk in the clinical manifestation of genetic FTLD. Systemic cardiovascular health is consistently among the most influential modifiable factors for all‐cause and sporadic dementia. Our study contributes to the existing literature on the strength of this heart‐to‐brain connection and extends these findings to middle‐aged adults at highest dementia risk and in FTLD specifically.

We found a beneficial relationship between cardiovascular health and memory and language trajectories in FTLD variant carriers that was not evident in cross‐sectional analyses. Our results differ from prior work in autosomal dominant forms of Alzheimer's disease (ADAD) and may reflect differences in FTLD versus AD progression.^31^ We did not detect a meaningful relationship between cardiovascular health and executive functioning in FTLD. While these results align with previous null associations evidenced in ADAD and CADASIL^1^ studies,^14^ we hypothesized a significant relationship between cardiovascular health and executive functioning, given the propensity for cerebrovascular injury to occur in anterior brain regions that support executive functioning.^32^ Perhaps cardiovascular health is not strong enough to overcome changes in one of the earliest, hallmark areas affected across FTLD syndromes.^20^ It is notable that cardiovascular health evidenced significantly stronger associations in FTLD variant carriers compared to non‐carriers. This suggests cardiovascular pathways may be particularly relevant in variant carriers, either via direct modulation of overlapping FTLD–cerebrovascular disease pathways or indirect promotion of protective pathways above and beyond FTLD biology. There is a growing body of literature demonstrating an interaction between AD pathology and cerebrovascular disease^33^, ^34^, ^35^; our work showing disproportionately stronger relationships between indicators of cardiovascular health and neurobehavioral outcomes in pathogenic mutation carriers suggests this may occur in FTLD spectrum disease as well. Given the heterogeneity of FTLD syndromes and systemic cardiovascular health, more work evaluating precise mechanistic targets is needed. In terms of cellular and molecular pathways underlying these cardiovascular relationships, immune or inflammatory factors may play an important role, consistent with increasing literature suggesting immune dysregulation impacts FTLD pathogenesis across genotypes.^36^, ^37^ As molecular biofluid markers become increasingly available, future human studies can begin to focus on disentangling the neurobiological pathways (e.g., angiogenic, inflammatory, lipid, etc.) that may be implicated in individuals with adverse systemic cardiovascular health profiles. To deploy precision medicine efforts, there is a need to delineate the pathways associated with cardiovascular health that may overlap with inherent degenerative risk in FTLD.

Beyond cognitive trajectories, we demonstrated an association between cardiovascular health and brain structural outcomes in FTLD variant carriers. Consistent with prior work, we showed a link between heart health and one of the current gold standard biomarkers of small vessel ischemic disease, WMHs, in individuals with FTLD. Though frontal white matter changes may also reflect neurodegenerative processes in FTLD, our data suggest that white matter changes do indeed associate with cardiovascular states in FTLD. Nonetheless, future work leveraging multimodal MRI markers of white matter health (e.g., diffusion tensor imaging, neurite orientation dispersion and density imaging, free water) and regionality of white matter changes would help better characterize the nature of how CVD may impact white matter integrity in FTLD variants. While we did not find a longitudinal relationship between LS7 and frontotemporal gray matter volume, we did find a positive cross‐sectional association. These latter findings are consistent with existing literature on genetic dementias which illustrate a baseline association between hippocampal gray matter atrophy and cardiovascular risk in CADASIL cross‐sectionally.^14^ We further build on these findings showing that protective associations between baseline cardiovascular health and frontotemporal volume are stronger in FTLD variant carriers compared to non‐carrier controls. This may suggest that cardiovascular health contributes to initial “brain reserve” (i.e., frontotemporal size), but does not influence the rate of gray matter atrophy over time in variant carriers.

The protective association between cardiovascular health and memory and language trajectories persisted in analyses restricted to asymptomatic variant carriers. These data support the clinical relevance of optimal cardiovascular health in individuals with genetic FTLD even before the overt manifestation of symptoms. Further, stratified models suggested that relationships between baseline cardiovascular health and cognitive trajectories were strongest in *MAPT* and *C9orf72* variant carriers. Of note, our sample of *GRN* variant carriers (*n* = 47) was the smallest. Alternatively, there could be a biological rationale explaining the lack of effects in *GRN* variant carriers, given prior work suggesting *GRN* carriers have more white matter disease that is not vascular in origin.^38^ Nonetheless, our data evidence positive cardiovascular‐related associations across genotypes reflecting both tau and TAR DNA‐binding protein 43 (TDP‐43) proteinopathies, suggesting benefits of systemic cardiovascular health in FTLD may not be specific to one type of proteinopathy.

In post hoc models, we demonstrated individual associations between blood pressure, cholesterol, body mass, and cognitive trajectories in variant carriers.^13^ These data contribute to the existing body of literature strongly supporting systolic blood pressure as an important, specific cardiovascular mechanism for healthy brain aging.^5^ In contrast, there is notable variability in existing literature supporting cholesterol and body size indices as protective for brain health. For instance, several meta‐analyses have demonstrated significant associations between higher total cholesterol and risk for all‐cause dementia, while many others did not demonstrate this significant association.^2^ Similarly, there is mixed support for body mass as an important factor for healthy brain aging. Some evidence supports midlife obesity as a risk factor for dementia, while other data suggest a significant reduction in risk for incident dementia in older adults with obesity.^39^ Future analyses integrating objective monitoring of cardiovascular health states (e.g., wearables) would help to more precisely understand optimal levels of cardiovascular health necessary to improve brain health trajectories in FTLD.

Our study is not without limitations. Given the observational study design, we cannot determine the directionality of associations between cardiovascular health and clinical outcomes. There have been no randomized clinical trials evaluating modifiable risk factors in FTLD, which would be needed to determine causal effects of cardiovascular health on brain and cognitive trajectories. Across cognitive domains, the participants who did not complete the composites were significantly older and had significantly higher CDR‐SB scores, as expected. Nonetheless, ability to participate in cognitive testing may bias our findings toward younger and less impaired participants. While LS7 includes both behavioral risk factors and health indicators, allowing for a holistic approach to cardiovascular health status,^15^ there are several self‐reported components in the metric (e.g., physical activity, diet) that carry inherent limitations of social and recall bias. Further, the ALLFTD study does not currently capture clinical labs that would improve the precision of the cardiovascular indicators (e.g., cholesterol, glucose). A deeper understanding of which aspects of cardiovascular health are most important for FTLD clinical outcomes is needed. Additionally, in a few of our analyses, our sample sizes were relatively small—that is, in longitudinal WMH analyses and within genotypes and individual LS7 components. Accordingly, it is possible we were underpowered to detect and precisely estimate gene‐specific relationships, as well as relationships related to individual LS7 components. Our study was limited by the inability to further validate these findings in a replication cohort and a relatively small sample size. As such, these data should be replicated in a larger sample to test the robustness of these relationships. Finally, the ALLFTD consortium is actively working toward a better characterization of this rare genetic disease, specifically in how it is represented across different ancestries and ethnicities. Nonetheless, the current study is limited by inadequate representation of ethnic/racial identities despite the ongoing work to enroll individuals in the ALLFTD study who have historically not been well‐represented in research.

These data are among the first to comprehensively examine several facets of cardiovascular health states in individuals with FTLD. Optimal cardiovascular health is an important, modifiable risk factor that demonstrates protective relationships with cognitive and WMH trajectories in FTLD variant carriers. Given that the behavioral symptoms associated with FTLD may predispose individuals to greater cardiovascular burden (e.g., apathy, hyperorality), understanding and intervening in this modifiable risk factor may be highly clinically relevant. Randomized controlled trials are warranted to explore the intersection between lifestyle and pharmacological interventions to attenuate FTLD disease progression. Continued examination of modifiable behaviors and cardiovascular health states in FTLD is needed to inform more precise recommendations, particularly those related to lifestyle interventions.

## CONFLICT OF INTEREST STATEMENT

Anna M. VandeBunte, Hyunwoo Lee, Emily W. Paolillo, Rowan Saloner, Katya Rascovsky, Allison Snyder, Mario F. Mendez, Ryan Darby, Hilary Heuer, and Kaitlin B. Casaletto have nothing to disclose. Ging‐Yuek Robin Hsiung received grant support from CIHR, NIH, and Alzheimer Society of BC; participated in clinical trials sponsored by Anavax, Biogen, Cassava, Eli Lilly, and Roche; and served as a consultant to Biogen, Novo Nordisk, and Roche. Adam Staffaroni is a co‐inventor of four ALLFTD mApp tasks and receives licensing fees. Datacubed Health was not involved with the analysis or reporting of study data. He has also received research support from the NIA/NIH, Bluefield Project to Cure FTD, the Alzheimer's Association, the Larry L. Hillblom Foundation, the Rainwater Charitable Foundation, and has provided consultation to Alector, Lilly/Prevail, Passage Bio, and Takeda. Carmela Tartaglia has served as an investigator for clinical trials sponsored by Biogen, Avanex, Green Valley, Roche/Genentech, Bristol Myers Squibb, Eli Lilly/Avid Radiopharmaceuticals, and Janssen. She receives research support from the Canadian Institutes of Health Research. Kristine Yaffe is on the board for Alector Inc. David S. Knopman serves on the data and safety monitoring board of the DIAN‐TU study; is a site principal investigator for clinical trials sponsored by Biogen, Lilly, and the University of Southern California; and is funded by the NIH. Eliana Marisa Ramos, Peter Pressman, and Julie A. Fields receive research support from NIH. Andrea C. Bozoki receives research funding from the NIH, Alector Inc., Cognition Therapeutics, EIP Pharma, and Transposon, Inc. She is a consultant for Eisai Pharmaceuticals and Creative Biopeptides and a member of the data safety monitoring board for AviadoBio. Bonnie Wong receives research support from the NIH. Kimiko Domoto‐Reilly receives research support from the NIH, and serves as an investigator for a clinical trial sponsored by Lawson Health Research Institute. Irene Litvan receives research support from NIH 1U19AG063911‐01 2R01AG038791‐06A, U01NS100610, U01NS80818, and 1R21NS114764‐01A1; the Michael J Fox Foundation, Parkinson Foundation, Lewy Body Association, CurePSP, Roche, Abbvie, Lundbeck, Novartis, Transposon, and UCB. She is a member of the scientific advisory board for the Rossy PSP Program at the University of Toronto, and of the scientific advisory board for Amydis but does not receive funds. She is the chief editor of *Frontiers in Neurology*. Douglas R. Galasko receives NIH research funding, clinical trial funding from Alector and Esai, and is a consultant to Esai, General Electric Health Care, Fujirebio, DSMB for Cyclo Therapeutics. Joseph C. Masdeu is a consultant and received research funding from Eli Lilly, parent co. of Avid Radiopharmaceuticals, manufacturer of 18F‐flortaucipir; receives personal fees from GE Healthcare; grants and personal fees from Eli Lilly; and grants from Acadia, Avanir, Biogen, Eisai, Janssen, NIH, Novartis, with no relation to the submitted work. Maria Belen Pasqual and Leah K. Forsberg receive research support from the NIH. Lawrence S. Honig receives research funding from Abbvie, Acumen, Alector, Biogen, BMS, Eisai, Genentech/Roche, Janssen/J&J, Transposon, UCB, Vaccinex. Consulting fees from Biogen, Cortexyme, Eisai, Medscape, and Prevail/Lilly. Nupur Ghoshal has participated or is currently participating in clinical trials of anti‐dementia drugs sponsored by the following companies: Bristol Myers Squibb, Eli Lilly/Avid Radiopharmaceuticals, Janssen Immunotherapy, Novartis, Pfizer, Wyeth, SNIFF (The Study of Nasal Insulin to Fight Forgetfulness) study, and A4 (The Anti‐Amyloid Treatment in Asymptomatic Alzheimer's Disease) trial. She receives research support from Tau Consortium and the Association for Frontotemporal Dementia and is funded by the NIH. Brian S. Appleby receives research support from CDC, NIH, Ionis, Alector, and the CJD Foundation. He has provided consultation to Acadia, Ionis, and Sangamo. Ian R. Mackenzie receives research funding from Canadian Institutes of Health Research, Alzheimer's Association US, NIH, and Weston Brain Institute. Joel H. Kramer receives research support from NIH and royalties from Pearson Inc. Adam Boxer is a co‐inventor of four of the ALLFTD mApp tasks and has previously received licensing fees. He also receives research support from the NIH (U19AG063911, R01AG038791, R01AG073482), the Tau Research Consortium, the Association for Frontotemporal Degeneration, Bluefield Project to Cure Frontotemporal Dementia, Corticobasal Degeneration Solutions, the Alzheimer's Drug Discovery Foundation, and the Alzheimer's Association. He has served as a consultant for Aeovian, AGTC, Alector, Arkuda, Arvinas, AviadoBio, Boehringer Ingelheim, Denali, GSK, Life Edit, Humana, Oligomerix, Oscotec, Roche, Transposon, TrueBinding and Wave, and received research support from Biogen, Eisai, and Regeneron. Brad Boeve has served as an investigator for clinical trials sponsored by Alector, Biogen, and Transposon. He receives royalties from the publication of a book entitled *Behavioral Neurology of Dementia* (Cambridge Medicine, 2009, 2017). He serves on the scientific advisory board of the Tau Consortium. He receives research support from the NIH, the Mayo Clinic Dorothy and Harry T. Mangurian Jr. Lewy Body Dementia Program, and the Little Family Foundation. Howard Rosen has received research support from Biogen Pharmaceuticals, has consulting agreements with Wave Neuroscience and Ionis Pharmaceuticals, and receives research support from NIH. Author disclosures are available in the supporting information.

## CONSENT STATEMENT

The study was approved by the appropriate institutional review boards and is conducted in accordance with the latest Declaration of Helsinki, including written informed consent from all participants.

## Supporting information

Supporting information

Supporting information

Supporting information

Supporting information

Supporting information

Supporting information

Supporting information

Supporting information

Supporting information

## Footnotes

1 cerebral autosomal dominant arteriopathy with subcortical infarcts and leukoencephalopathy
