# Supplementary material for: Better cardiovascular health is associated with slowed clinical progression in autosomal dominant frontotemporal lobar degeneration variant carriers
Source: Alzheimers Dement. 2024 Sep 6;20(10):6820–33. doi: 10.1002/alz.14172 (PMC11485313; doi:10.1002/alz.14172)
Supplement: Supplementary file 1 — Supporting information [file ALZ-20-6820-s003.docx]

**Supplemental Figure 1.** Linear mixed-effects models examining associations between individual Life’s Simple 7 factors and memory trajectories in FTLD variant carriers. Accounts for baseline age, sex, education, and FTLD-CDR.
