# Supplementary material for: Better cardiovascular health is associated with slowed clinical progression in autosomal dominant frontotemporal lobar degeneration variant carriers
Source: Alzheimers Dement. 2024 Sep 6;20(10):6820–33. doi: 10.1002/alz.14172 (PMC11485313; doi:10.1002/alz.14172)
Supplement: Supplementary file 2 — Supporting information [file ALZ-20-6820-s009.docx]

**Supplemental Figure 2.** Linear mixed-effects models examining associations between individual Life’s Simple 7 factors and language trajectories in FTLD variant carriers. Accounts for baseline age, sex, education, and FTLD-CDR.

<< PRINT IN COLOR >>
