# Supplementary material for: Better cardiovascular health is associated with slowed clinical progression in autosomal dominant frontotemporal lobar degeneration variant carriers
Source: Alzheimers Dement. 2024 Sep 6;20(10):6820–33. doi: 10.1002/alz.14172 (PMC11485313; doi:10.1002/alz.14172)
Supplement: Supplementary file 4 — Supporting information [file ALZ-20-6820-s005.docx]

**Supplemental Table 1.** Breakdown of clinical phenotypes by genotype in baseline sample of symptomatic variant carriers.

|  | ***C9orf72***  **(N = 71)** | ***GRN***  **(n=22)** | ***MAPT***  **(n=23)** |
| --- | --- | --- | --- |
|  |  |  |  |
| Clinical Phenotype  (%, n)  MCI* cognitive variant  MCI* language variant  MCI* behavior variant  Semantic variant PPA+  Agrammatic/non-fluent variant PPA+  Behavioral variant FTD†  Corticobasal syndrome  FTD†/Amyotrophic lateral sclerosis  Amyotrophic lateral sclerosis  PSP°/Richardson’s syndrome  Alzheimer’s disease dementia  Dementia with Lewy bodies  Other± | 14.1% (10)  0% (0)  0% (0)  1.4% (1)  1.4% (1)  54.9% (39)  1.4% (1)  8.5% (6)  8.5% (6)  2.8% (2)  0% (0)  0% (0)  7.0% (5) | 0% (0)  0% (0)  4.5% (1)  0% (0)  4.5% (1)  54.5% (12)  22.7% (5)  0% (0)  0% (0)  0% (0)  4.5% (1)  4.5% (1)  4.5% (1) | 13.0% (3)  4.3% (1)  4.3% (1)  4.3% (1)  0% (0)  65.2% (15)  0% (0)  0% (0)  0% (0)  0% (0)  8.7% (2)  0% (0)  0% (0) |

**Note.** *Mild cognitive impairment. +Primary progressive aphasia. †Frontotemporal dementia. °Progressive supranuclear palsy. Three participants who carry both *C9orf72* and *GRN* variants were excluded from the table. ±Participants in this category had diagnoses related to neurodevelopmental, substance use, or other factors (obsessive compulsive disorder, nonspecific PPA).
