# Supplementary material for: Better cardiovascular health is associated with slowed clinical progression in autosomal dominant frontotemporal lobar degeneration variant carriers
Source: Alzheimers Dement. 2024 Sep 6;20(10):6820–33. doi: 10.1002/alz.14172 (PMC11485313; doi:10.1002/alz.14172)
Supplement: Supplementary file 5 — Supporting information [file ALZ-20-6820-s007.docx]

**Supplemental Table 2**. Linear regression two-way interaction models (Carrier Status*LS7) examining differences in associations between baseline LS7 and cognitive and brain structural outcomes based on variant carrier status.

|  | **Memory** | | **Language** | | | **Executive Functioning** | | | **Frontotemporal GMV** | | | | **Frontal WMH** | |
| --- | --- | --- | --- | --- | --- | --- | --- | --- | --- | --- | --- | --- | --- | --- |
|  | **β (95% CI)** | **p-value** | | **β (95% CI)** | **p-value** | | **β (95% CI)** | **p-value** | | **β (95% CI)** | **p-value** | **β (95% CI)** | | **p-value** |
| LS7  (0-14) | 0.03  (-0.07, 0.14) | 0.560 | | 0.08  (-0.03, 0.19) | 0.154 | | 0.03  (-0.08, 0.13) | 0.620 | | -0.02  (-0.11, 0.06) | 0.601 | -0.07  (-0.24, 0.11) | | 0.455 |
| Carrier Status | -0.19  (-0.33, -0.04) | 0.010* | | -0.14  (-0.29, 0.01) | 0.060 | | -0.17  (-0.31, -0.02) | 0.020* | | -0.21  (-0.33,  -0.10) | <0.001* | 0.06  (-0.18, 0.29) | | 0.395 |
| Age | -0.18  (-0.26, -0.11) | <0.001* | | 0.06  (-0.02, 0.14) | 0.144 | | -0.04  (-0.11, 0.04) | 0.329 | | -0.43  (-0.49,  -0.37) | <0.001* | 0.37  (0.24, 0.49 | | <0.001* |
| Education | 0.14  (0.07, 0.21) | <0.001* | | -0.06  (-0.13, 0.01) | 0.096 | | 0.03  (-0.04, 0.10) | 0.402 | | -0.02  (-0.08, 0.04) | 0.514 | -0.08  (-0.20, 0.04) | | 0.197 |
| Sex | 0.04  (-0.09, 0.18) | 0.513 | | 0.14  (0.00, 0.28) | 0.043* | | 0.08  (-0.06, 0.21) | 0.267 | | 0.12  (-0.03, 0.27) | 0.106 | 0.03  (-0.24, 0.30) | | 0.813 |
| CDR®+NACC FTLD-SB | -0.56  (-0.64, -0.48) | <0.001* | | -0.70  (-0.78, -0.62) | <0.001* | | -0.66  (-0.73, -0.58) | <0.001* | | -0.35  (-0.41,  -0.29) | <0.001* | 0.16  (0.10, 0.35) | | <0.001* |
| Total Intracranial volume | - | - | | - | - | | - | - | | 0.55  (0.48, 0.63) | <0.001* | 0.01  (-0.13, 0.14) | | 0.892 |
| LS7*Carrier Status | -0.01  (-0.15, 0.12) | 0.828 | | -0.07  (-0.21, 0.07) | 0.309 | | 0.03  (-0.11, 0.16) | 0.670 | | 0.18  (0.07, 0.29) | 0.001* | 0.12  (-0.11, 0.34) | | 0.315 |

**Note.** β standardized beta values; CDR®+NACC FTLD-SB = CDR Dementia Staging Instrument PLUS National Alzheimer’s Coordinating Center (NACC) Behavior and Language Domain, sum of boxes; LS7 = Life’s Simple 7, where higher scores represent more optimal cardiovascular health.
