# Supplementary material for: Better cardiovascular health is associated with slowed clinical progression in autosomal dominant frontotemporal lobar degeneration variant carriers
Source: Alzheimers Dement. 2024 Sep 6;20(10):6820–33. doi: 10.1002/alz.14172 (PMC11485313; doi:10.1002/alz.14172)
Supplement: Supplementary file 6 — Supporting information [file ALZ-20-6820-s001.docx]

**Supplemental Table 3**. Sensitivity mixed effects models examining associations between baseline LS7 and cognitive (memory and language) and frontal white matter hyperintensity trajectories in variant carriers only, accounting for age*time, sex*time, education*time

|  | **Memory** | | **Language** | | **Frontal WMH** | |
| --- | --- | --- | --- | --- | --- | --- |
|  | **β (95% CI)** | **p-value** | **β (95% CI)** | **p-value** | **β (95% CI)** | **p-value** |
| Baseline age | -0.34  (-0.47,  -0.21) | <0.001* | -0.04  (-0.19, 0.10) | 0.54 | 0.45  (0.24, 0.65) | <0.001* |
| Education | 0.11  (-0.01, 0.22) | 0.07 | -0.08  (-0.20, 0.05) | 0.22 | -0.15  (-0.35, 0.04) | 0.13 |
| Sex | -0.07  (-0.29, 0.15) | 0.53 | 0.13  (-0.11, 0.37) | 0.30 | -0.01  (-0.49, 0.47) | 0.97 |
| Baseline CDR®+NACC FTLD-SB | -0.49  (-0.59,  -0.40) | <0.001* | -0.71  (-0.82,  -0.59) | <0.001* | 0.23  (0.04, 0.42) | 0.02* |
| Baseline total intracranial volume | - | - | - | - | -0.05  (-0.28, 0.19) | 0.70 |
| Baseline LS7 (0-14) | 0.03  (-0.09, 0.15) | 0.64 | 0.01  (-0.12, 0.14) | 0.88 | -0.02  (-0.23, 0.19) | 0.84 |
| Time in study | 0.02  (-0.07, 0.11) | 0.68 | -0.07  (-0.15, 0.00) | 0.06 | -0.02  (-0.18, 0.15) | 0.83 |
| Baseline age*Time | -0.14  (-0.20,  -0.08) | <0.001***** | -0.10  (-0.15,  -0.05) | <0.001* | -0.09  (-0.20, 0.03) | 0.15 |
| Education*Time | 0.00  (-0.06, 0.06) | 0.93 | 0.04  (-0.02, 0.09) | 0.18 | 0.03  (-0.09, 0.14) | 0.66 |
| Sex*Time | -0.03  (-0.14, 0.09) | 0.67 | 0.03  (-0.08, 0.13) | 0.62 | 0.12  (-0.09, 0.32) | 0.28 |
| Baseline LS7*Time | 0.06  (-0.01, 0.12) | 0.09 | 0.05  (-0.01, 0.10) | 0.09 | -0.13  (-0.23,  -0.02) | 0.03* |

**Note.** β standardized beta values; CDR®+NACC FTLD-SB = CDR Dementia Staging Instrument PLUS National Alzheimer’s Coordinating Center (NACC) Behavior and Language Domain, sum of boxes; LS7 = Life’s Simple 7, where higher scores represent more optimal cardiovascular health.
