# Supplementary material for: Better cardiovascular health is associated with slowed clinical progression in autosomal dominant frontotemporal lobar degeneration variant carriers
Source: Alzheimers Dement. 2024 Sep 6;20(10):6820–33. doi: 10.1002/alz.14172 (PMC11485313; doi:10.1002/alz.14172)
Supplement: Supplementary file 7 — Supporting information [file ALZ-20-6820-s004.docx]

**Supplemental Table 4**. Sensitivity mixed effects three-way interaction model examining the difference in the association between baseline LS7 and memory trajectories based on variant carrier status, accounting for age*time, sex*time, education*time

|  | **Memory** | |
| --- | --- | --- |
|  | **β (95% CI)** | **p-value** |
| Time in study | 0.16 (0.07, 0.24) | <0.001* |
| Baseline LS7 (0-14) | 0.05 (-0.07, 0.17) | 0.41 |
| Carrier Status (non-carrier /variant carrier) | -0.30 (-0.47, -0.13) | <0.001* |
| Baseline age | -0.24 (-0.33, -0.15) | <0.001* |
| Education | 0.08 (0.00, 0.17) | 0.025* |
| Sex | 0.03 (-0.14, 0.19) | 0.75 |
| Baseline CDR®+NACC FTLD-SB | -0.48 (-0.56, -0.41) | <0.001* |
| Baseline LS7*Time | -0.02 (-0.09, 0.05) | 0.51 |
| Carrier Status*Time | -0.15 (-0.24, -0.06) | <0.001* |
| Baseline age*Time | -0.08 (-0.13, -0.04) | <0.001* |
| Education*Time | -0.01 (-0.06, 0.03) | 0.57 |
| Sex*Time | 0.01 (-0.08,0.10) | 0.80 |
| Baseline LS7*Carrier Status | 0.02 (-0.14, 0.18) | 0.84 |
| Baseline LS7*Carrier Status*Time | 0.11 (0.02, 0.20) | 0.022* |

**Note.** β standardized beta values; CDR®+NACC FTLD-SB = CDR Dementia Staging Instrument PLUS National Alzheimer’s Coordinating Center (NACC) Behavior and Language Domain, sum of boxes; LS7 = Life’s Simple 7, where higher scores represent more optimal cardiovascular health.
