# Supplementary material for: Better cardiovascular health is associated with slowed clinical progression in autosomal dominant frontotemporal lobar degeneration variant carriers
Source: Alzheimers Dement. 2024 Sep 6;20(10):6820–33. doi: 10.1002/alz.14172 (PMC11485313; doi:10.1002/alz.14172)
Supplement: Supplementary file 8 — Supporting information [file ALZ-20-6820-s002.docx]

**Supplemental Table 5**. Mixed effects three-way interaction models examining differences in associations between baseline LS7 and cognitive and brain structural trajectories based on variant carrier status.

|  | **Memory** | | **Language** | | **Executive Functioning** | | **Frontotemporal GMV** | | **Frontal WMH** | |
| --- | --- | --- | --- | --- | --- | --- | --- | --- | --- | --- |
|  | **β (95% CI)** | **p-value** | **β (95% CI)** | **p-value** | **β (95% CI)** | **p-value** | **β (95% CI)** | **p-value** | **β (95% CI)** | **p-value** |
| Time in study | 0.17 (0.10,  0.23) | 0.293 | 0.08 (0.03, 0.14) | 0.953 | 0.10  (0.03, 0.16) | 0.787 | -0.08 (-0.11, -0.04) | 0.526 | -0.05 (-0.16, 0.05) | 0.862 |
| Baseline LS7 (0-14) | 0.07  (-0.05, 0.19) | 0.399 | 0.08  (-0.04, 0.21) | 0.521 | 0.12  (0.00, 0.24) | 0.164 | -0.04 (-0.14, 0.07) | 0.627 | 0.04  (-0.15, 0.24) | 0.544 |
| Carrier Status (non-carrier /variant carrier) | -0.29 (-0.46, -0.11) | 0.115 | -0.18  (-0.36, -0.00) | 0.049* | -0.20  (-0.38, -0.03) | 0.171 | -0.26 (-0.41, -0.11) | 0.001* | 0.02  (-0.27, 0.30) | 0.506 |
| Baseline age | -0.18 (-0.27, -0.10) | <0.001* | 0.02  (-0.07, 0.11) | 0.691 | 0.03  (-0.06, 0.11) | 0.550 | -0.51 (-0.59, -0.43) | <0.001* | 0.44 (0.29, 0.59) | <0.001* |
| Education | 0.09 (0.01, 0.17) | 0.022* | -0.06  (-0.14, 0.03) | 0.199 | 0.02  (-0.06, 0.09) | 0.703 | -0.03 (-0.10, 0.04) | 0.412 | -0.14 (-0.29, 0.00) | 0.058 |
| Sex | 0.02  (-0.14, 0.18) | 0.825 | 0.15  (-0.02, 0.32) | 0.090 | -0.05  (-0.21, 0.11) | 0.534 | 0.17  (-0.03, 0.37) | 0.096 | -0.06 (-0.40, 0.28) | 0.733 |
| Baseline CDR®+NACC FTLD-SB | -0.48  (-0.56, -0.41) | <0.001* | -0.68  (-0.76, -0.60) | <0.001* | -0.62  (-0.69, -0.54) | <0.001* | -0.25 (-0.31, -0.19) | <0.001* | 0.18 (0.04, 0.32) | 0.013* |
| Baseline total intracranial volume | - | - | - | - | - | - | 0.56 (0.46, 0.65) | <0.001* | 0.02  (-0.15, 0.19) | 0.802 |
| Baseline LS7*Time | 0.00  (-0.06, 0.07) | 0.908 | 0.02  (-0.04, 0.08) | 0.533 | 0.01  (-0.05, 0.08) | 0.679 | -0.01 (-0.04, 0.03) | 0.719 | -0.02 (-0.12, 0.08) | 0.668 |
| Carrier Status*Time | -0.14  (-0.22,  -0.05) | 0.007* | -0.13  (-0.20, -0.06) | 0.015* | -0.12  (-0.21, -0.04) | 0.117 | -0.06 (-0.11, -0.02) | 0.945 | 0.12  (-0.02, 0.25) | 0.161 |
| Baseline LS7*Carrier Status | 0.01  (-0.15, 0.17) | 0.081 | -0.07  (-0.23, 0.09) | 0.056 | -0.05  (-0.21, 0.12) | 0.170 | 0.18 (0.04, 0.32) | 0.005* | -0.07 (-0.35, 0.21) | 0.677 |
| Baseline LS7*Carrier Status*Time | 0.10  (0.01,  0.18) | 0.035* | 0.07  (-0.01, 0.15) | 0.079 | 0.04  (-0.04, 0.13) | 0.334 | -0.02 (-0.06, 0.03) | 0.490 | -0.07 (-0.21, 0.06) | 0.290 |

**Note.** β standardized beta values; CDR®+NACC FTLD-SB = CDR Dementia Staging Instrument PLUS National Alzheimer’s Coordinating Center (NACC) Behavior and Language Domain, sum of boxes; LS7 = Life’s Simple 7, where higher scores represent more optimal cardiovascular health.
